# Genome-Wide Identification and Characterization of the Shaker-Type K^+^ Channel Genes in *Prunus persica* (L.) Batsch

**DOI:** 10.1155/2022/5053838

**Published:** 2022-03-09

**Authors:** Yong Yang, Jinlong Han, Yue Zhang, Shizhuo Lin, Meixia Liang, Lizi Zhao, Zhizhong Song

**Affiliations:** ^1^Zhenjiang Academy of Agricultural Sciences, Zhenjiang Institute of Agricultural Sciences in Hilly Areas of Jiangsu Province, Zhenjiang 212400, China; ^2^The Engineering Research Institute of Agriculture and Forestry, Ludong University, Yantai 264025, China; ^3^Nanjing Academy of Agricultural Sciences, Nanjing 214000, China; ^4^College of Forestry, Nanjing Forestry University, Nanjing 210037, China

## Abstract

Shaker-type K^+^ channels are critical for plant K^+^ acquisition and translocation that play key roles during plant growth and development. However, molecular mechanisms towards K^+^ channels are extremely rare in fruit trees, especially in peach. In this study, we identified 7 putative shaker-type K^+^ channel genes from peach, which were unevenly distributed on 5 chromosomes. The peach shaker K^+^ channel proteins were classified into 5 subfamilies, I-V, and were tightly clustered with pear homologs in the phylogenetic tree. Various *cis*-acting regulatory elements were detected in the promoter region of the shaker-type K^+^ channel genes, including phytohormone-responsive, abiotic stress-responsive, and development regulatory elements. The peach shaker K^+^ channel genes were expressed differentially in distinct tissues, and *PpSPIK* was specifically expressed in the full-bloom flowers; *PpKAT1* and *PpGORK* were predominantly expressed in the leaves, while *PpAKT1*, *PpKC1*, and *PpSKOR* were majorly expressed in the roots. The peach shaker K^+^ channel genes were differentially regulated by abiotic stresses in that K^+^ deficiency, and ABA treatment mainly increased the shaker K^+^ channel gene expression throughout the whole seedling, whereas NaCl and PEG treatment reduced the shaker K^+^ channel gene expression, especially in the roots. Moreover, electrophysiological analysis demonstrated that PpSKOR is a typical voltage-dependent outwardly rectifying K^+^ channel in peach. This study lays a molecular basis for further functional studies of the shaker-type K^+^ channel genes in peach and provides a theoretical foundation for K^+^ nutrition and balance research in fruit trees.

## 1. Introduction

Potassium (K^+^) is an essential macronutrient for plants to maintain crucial roles in a number of biochemical and physiological processes [1–3]. Xylem is the transport organization of vascular plants, which is responsible for the upward transport of K^+^ absorbed by the roots. Phloem is a complex tissue that transports, supports, and stores nutrients, including K^+^, especially in ferns and seed plants [1, 2, 4]. The K^+^ from the soil solution was taken up via the root's surface and then transported to the shoots, distributed within cells into different compartments, and recycled in storage organs by various K^+^ transport systems, including the shaker-type K^+^ channels, KT/HAK/KUP transporters, tandem-pore K^+^ (TPK) channels, and cation-proton antiporters (CPAs) [2, 4, 5].

In plants, there are two kinds of K^+^ uptake and transport mechanisms, i.e., the high-affinity K^+^ absorption system (mechanism I) and the low-affinity K^+^ absorption system (mechanism II). The mechanism I system plays a crucial role just when the external K^+^ status is less than 200 *μ*mol·L^−1^, while the mechanism II system plays an important role when the external K^+^ status is more than 1 mmol·L^−1^ [6, 7]. In particular, the long-distance K^+^ distribution and dynamic balance are mainly mediated by 3 categories of K^+^ channels, including shaker-type channels, TPK family channels, and other K^+^ channels, which have been functionally verified via electrophysiological systems [2, 4, 5, 8, 9]. Notably, shaker K^+^ channels were the first K^+^ channels identified in plants at the molecular level [10]. According to the voltage dependence and K^+^ movement direction, there are 9 shaker-like K^+^ channels in *Arabidopsis*, including the inward-rectifying K^+^ channels AtKAT1, AtKAT2, AtAKT1, AtAKT5, and AtSPIK, the weak-rectifying K^+^ channel AtAKT2, the outward-rectifying K^+^ channels AtSKOR and AtGORK, and regulatory subunit AtKC1 [1, 8, 11–21]. Several members of the shaker K^+^ channel gene family have been cloned and functionally determined by heterologous expression system or electrophysiological system from tomato [12, 13], barley [14], maize [15], rice [16], carrot [17], *Ammopoptanthus mongolicus* [18], grape [19–21], strawberry [11], pear [8], and osier willow [9].

Peach (*Prunus persica* (L.) Batsch) is one of the most important fruit crops in the world [22]. K^+^ is the most abundant cation within the fruit that plays an important role in all developmental stages, and K^+^ deficiency negatively affects fruit productivity and fruit quality [23–25]. However, molecular mechanism towards K^+^ transport and distribution in fruits is unclear. In this study, 7 putative shaker-type K^+^ channel genes were identified in peach, and the detailed gene location, phylogenetic relationships, gene structures, and tissue expression profiles were further investigated. This study provides a foundation for further functional characterization of the shaker-like K^+^ channels in peach.

## 2. Materials and Methods

### 2.1. Identification and Classification of Putative Peach Shaker K^+^ Channel Genes

Peach genome datasets were downloaded from the Phytozome v 13 peach genome database (http://phytozome-next.jgi.doe.gov). The protein sequences of the 9 shaker K^+^ channel genes of *Arabidopsis* were obtained from the Arabidopsis Information Resource (TAIR) (http://www.arabidopsis.org). BLASTP searches against the peach genome database were performed using the full-length sequences of *Arabidopsis* shaker K^+^ channel proteins as queries. To confirm the existence of the shaker K^+^ channel protein domains (PF00027, PF00520, and PF11834) [7–9], the candidate proteins were analyzed using Pfam (http://pfam.xfam.org) and Simple Modular Architecture Research Tool (http://smart.embl-heidelberg.de/). To distinguish the candidate peach shaker K^+^ channel genes, we entitled them in accordance with the order of the corresponding phylogenetic locations. The molecular weights, isoelectric points (pI), aliphatic index, and grand average of hydropathicity (GRAVY) of the peach shaker K^+^ channel proteins were calculated by the ExPasy website (https://web.expasy.org/protparam/). The subcellular locations of the peach shaker K^+^ channel proteins were predicted by WoLF PSORT (http://www.genscript.com/psort/wolf_psort.html). The exon-intron structure was determined using the online program Gene Structure Display Server: GSDS 2.0 (http://gsds.gao-lab.org/), and transmembrane domains were predicted by the online program TMpredict (http:// http://sbcb.bioch.ox.ac.uk/TM_noj/TM_noj.html).

### 2.2. Phylogenetic Tree Construction of Plant Shaker K^+^ Channel Homologs

A multiple alignment analysis among the shaker K^+^ channel homologs from peach, *Arabidopsis*, rice, pear, sorghum, and maize was carried out using the ClustalW software. Gene ID of the shaker K^+^ channel homologs are listed in Supplemental Table 1. The phylogenetic tree was generated using MEGA13.0 with the maximum likelihood (ML) method, and the bootstrap analysis was set to 1000 replicates.

### 2.3. Cis-acting Element Prediction of the Promoter Regions of Peach Shaker K^+^ Channel Genes

The 1500 bp upstream sequence of coding regions of the shaker K^+^ channel genes were retrieved from the Phytozome peach genome database and then submitted to PlantCARE (http://bioinformatics.psb.ugent.be/webtools/plantcare/html/).

### 2.4. Plant Materials and Treatments

Five-year-old *Prunus persica* (L.) Batsch cv. Xiahui 6 trees growing at the Jiangsu Peach Germplasm Repository (Zhenjiang China) were used throughout this study. The leave, phloem, flower and fruit samples were collected at different developmental stages (DS), as described in our previous reports [26–28]. For stress treatments, Xiahui 6 seedlings were germinated from seeds on MS solid medium and cultured in the incubator of 28°C day 16 h/18°C night 8 h, with a relative humidity of 75%, for 4 weeks, and then treated by K^+^ depletion, 200 *μ*mol·L^−1^ ABA, 200 mmol·L^−1^ NaCl, or 10% (w/v) PEG for 48 h, respectively [9, 29–31]. The MS medium was used as a control. The samples were frozen in liquid nitrogen and stored at -80°C for RNA extraction and gene expression analysis.

### 2.5. Quantitative Real-Time PCR (RT-qPCR)

The total RNA of each sample was extracted using MiniBEST Plant RNA extraction kit (TaKaRa, Dalian, China), and the 1st-strand cDNA was synthesized using Primer Script RT reagent kit (TaKaRa, Dalian, China). Specific primers were designed using the NCBI Primer BLAST online tool against the peach genome (Supplemental Table 2). The qRT-PCR analysis was performed on 7500 Real-Time PCR System (Applied Biosystems, New York, USA) using SYBR Green (TaKaRa, Dalian, China). The peach *UBI* gene was used as the internal control [26–29, 31]. The RT-qPCR reaction procedure was as follows: 95°C for 30 sec, 40 cycles of 95°C for 5 sec, and 60°C for 34 s, and then 72°C for 60 sec. All reactions were performed in triplicates, and three biological repeats were conducted. The relative transcript level of each gene is calculated using the 2^*−Δ*CT^ normalized expression method [26–29, 31].

### 2.6. Patch Clamping Analysis

The electrophysiological function of *PpSKOR* was verified by patch clamping system as described previously [9, 30]. The expression plasmid pTracer-CMV3-*SKOR* was constructed by introducing the *PpSKOR* gene into the vector of pTracer-CMV3 [9, 30]. The primers used for the recombinant vector construction are listed in Supplemental Table 2, and *Pme* I site was introduced in the forward primer, and *Not* I site was introduced in the reverse primer, which were both underlined. The HEK293-T cells transfected with pTracer-CMV3 empty vector were used as the control, and pCLAMP 10.0 patch clamping system was utilized to record the currents of pTracer-CMV3-*SKOR* under different extracellular K^+^ concentrations [9, 30], including 0, 10, 50, and 100 mmol·L^−1^, without deducting the control background currents.

### 2.7. Statistical Analysis

Statistical analysis was carried out using independent samples *t* test in SPSS 22.0 software (SPSS Chicago, Illinois, USA). *Asterisks* indicate statistical differences between plants under control and stress treatment (^∗^*P* < 0.05, and ^∗∗^*P* < 0.01; independent samples *t* test).

## 3. Results

### 3.1. Genome-Wide Identification of the Shaker K^+^ Channel Genes in Peach

**Figure 1:**
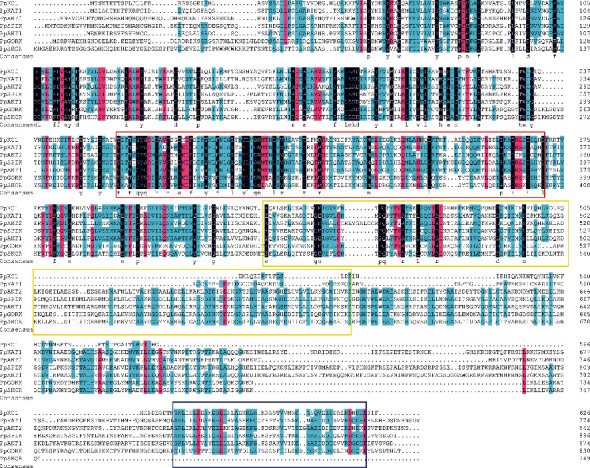
Multiple sequence analysis of the peach shaker K^+^ channel proteins.

**Figure 2:**
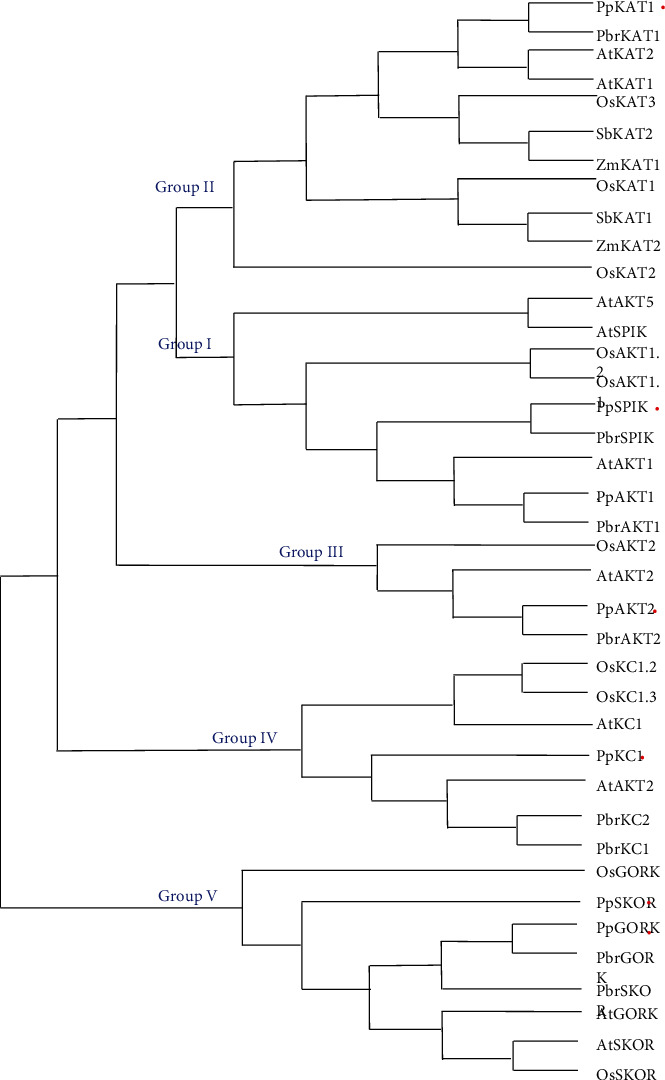
Phylogenetic tree of the shaker K^+^ channel proteins from different plants.

In this present study, a total number of 7 nonredundant shaker K^+^ channel genes were screened and identified from peach genome (Table 1). Functional domain verification and multiple sequence analysis showed that all peach shaker K^+^ channel proteins contained the cyclic nucleotide-binding domain (PF00027), ion channel transmembrane (PF00520), and KHA domain (PF11834), which belonged to the classic plant shaker K^+^ channels (Figure 1). To further entitle the peach shaker K^+^ channel genes with individual names and investigate the evolutionary relationship of the plant shaker channel homologs, a ML phylogenetic tree was constructed among peach, pear, *Arabidopsis*, rice, sorghum, and maize (Figure 2). Notably, the amino acid sequences of the shaker K^+^ channel proteins from these 6 plant species shared an overall identity of 65.13%, and the highest identity (86.51%) was observed in extremely conserved domains or regions (Supplemental Figure 1). According to the tree, the plant shaker channel homologs could be divided into 5 subfamilies, including group I-V, and the peach shaker K^+^ channel proteins were distributed in group I-V subfamilies, each with 2, 1, 1, 1, and 2 members, respectively (Figure 2). In particular, all peach shaker channel proteins were strictly clustered with corresponding homologs from pear, with the exception of PpSKOR that was clustered among SKOR or GORK homologs from different plant species (Figure 2).

Multiple alignment of the peach shaker K^+^ channel proteins was analyzed using ClustalX2.1 software. The peach shaker K^+^ channel proteins were labelled with red dot. The locations of the functional domains were labelled with squares of different colors (PF00027, cyclic nucleotide-binding domain, red square; PF00520, ion channel transmembrane, blue square; and PF11834, KHA domain, yellow square).

A maximum likelihood (ML) tree was constructed by multiple alignment of the shaker K^+^ channel proteins in peach, pear, *Arabidopsis*, rice, soybean, and maize using ClustalX2.1 and MEGA13.0 software. The information of the shaker K^+^ channel proteins from the sequenced plant was listed in Supplemental Table 1. The peach shaker K^+^ channel proteins were labelled with red dot.

### 3.2. Bioinformatic Analysis of the Peach Shaker K^+^ Channel Genes

**Figure 3:**
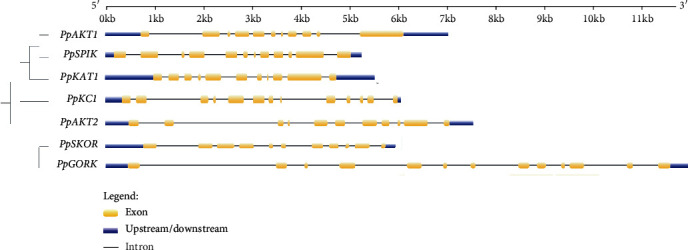
Gene structure analysis of the peach shaker K^+^ channel genes.

The basic information of the peach shaker K^+^ channel genes is listed in Table 1, including the identity number, chromosome distribution, gene location, and intron numbers. In general, the peach shaker K^+^ channel genes were distributed in 5 distinct chromosomes (Chr1, 3, 4, 5, and 7), each with 3, 1, 1, 1, and 1 gene. Notably, PpSPIK (group I), PpAKT2 (group III), and PpKC1 (group IV) belonging to different subfamilies were located in the same Chr1, while PpSPIK and PpAKT1 belonging to group I were distributed in different chromosomes. The gene structure analysis showed that the peach shaker K^+^ channel genes possessed 10, 11, 10, 12, 10, 10, and 12 introns, respectively, that varied distinctly in length (Figure 3 and Table 1). In particular, *PpGORK* had the largest intron (>2.7 kb) and *PpKC1* possessed the shortest intron (<70 bp).

According to the value of theoretical pI, all peach shaker K^+^ channel proteins were acidic amino acids except for PpAKT2 (from group V) that was alkalescent (Table 1). Transmembrane (TM) prediction revealed that all peach shaker channel proteins possessed 6 TM domains, which is the same as in the previous reports [3, 7, 8, 30]. Moreover, GRAVY index analysis showed that all peach shaker channel proteins were hydrophilic proteins (<0), except for PpAKT1 (0.728) that was a hydrophobic protein (Table 1). The aliphatic index analyses indicated that all peach shaker channel proteins had low values that are less than 100, except for PpAKT1 (127.72), which again supports the predication that these channel proteins are hydrophilic proteins.

Subcellular localization prediction indicated that PpAKT1 was totally localized in the plasma membrane, and the other 5 channel proteins are majorly (at least 50%) localized in plasma membrane, followed by endoplasmic reticulum membrane and cytosol (Table 2). In addition, 3, 3, and 2 channels were also observed in the microbody, nucleus, and mitochondrial inner membrane, respectively. PpGORK was also detected in the chloroplast membrane and Golgi body (Table 2).

### 3.3. Analyses of Cis-acting Elements of Peach Shaker K^+^ Channel Genes

Prediction results showed that the peach shaker channel genes harbored at least 16 kinds of c*is*-acting elements in their promoter regions, including 6 kinds of stress-responsive, 5 kinds of hormone-responsive, and 5 kinds of metabolism and development regulatory elements, with different existence numbers (Table 3). Notably, each peach shaker channel gene possessed at least 7 kinds of *cis*-acting elements, and all peach shaker channel genes possessed light responsive (at least 6), anaerobic induction (1), and abscisic acid-responsive elements (1). In addition, there were at least 3 hormone-responsive elements that could be observed in all peach shaker channel genes, with the exception of *PpAKT1* that just had abscisic acid-responsive element. And there was at least 1 stress-responsive and 1 metabolism and development regulatory element that could be observed in all peach shaker channel genes (Table 3).

### 3.4. Tissue-Specific Expression Pattern Analysis of the Peach Shaker K^+^ Channel Genes

**Figure 4:**
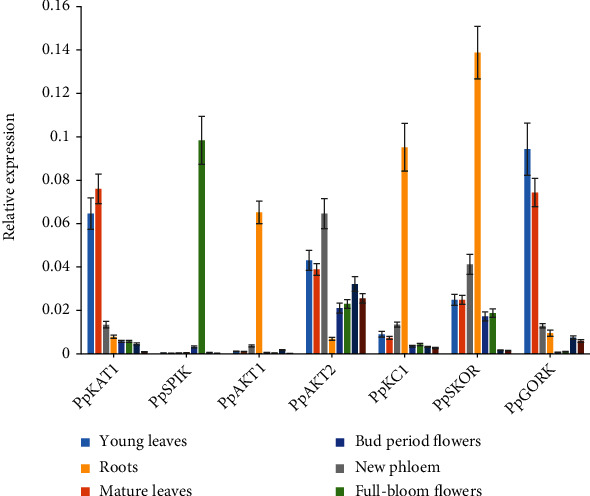
Tissue-/organ-specific expression analysis of the peach shaker K^+^ channel genes.

To further illustrate the potential functions in peach, the expression profiles of the shaker channel genes were analyzed via RT-qPCR in different tissues or organs in 5-year-old peach trees. The results showed that the shaker K^+^ channel genes exhibited distinct tissue-specific characteristics in peach trees (Figure 4). In particular, *PpSPIK* was specifically expressed in the full-bloom flowers, *PpKAT1* and *PpGORK* were predominantly expressed in the leaves, while *PpAKT1*, *PpKC1*, and *PpSKOR* were majorly expressed in the roots (Figure 4). Notably, the expression of *PpAKT2* was higher and relatively even in the aboveground parts than in the roots, and the highest level was observed in the phloem. The distinct tissue-specific expression profiles may reflect different channel functions that taken place in special parts of peach trees.

### 3.5. Response of the Peach Shaker K^+^ Channel Genes under Abiotic Stresses

**Figure 5:**
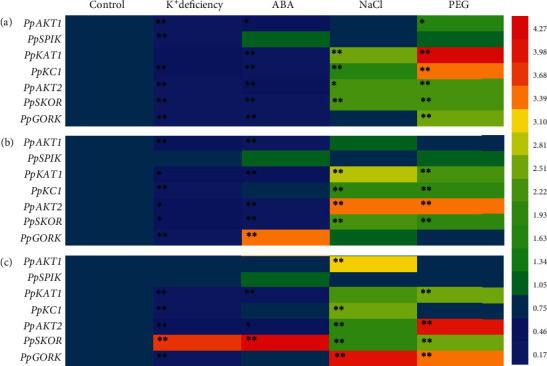
Heat map analysis of the peach shaker K^+^ channel genes in response to K^+^ depletion, ABA, NaCl, and PEG stresses in seedlings. *Asterisks* indicate statistical differences between plants under control and stress treatment (^∗^*P* < 0.05 and ^∗∗^*P* < 0.01; independent samples *t* test).

We further examined the relative expression levels of the peach shaker K^+^ channel genes in peach seedlings in response to abiotic stresses, including K^+^ deficiency, NaCl, ABA, and PEG treatment, respectively. In general, the RT-qPCR indicated that the shaker K^+^ channel genes were differentially regulated by these abiotic stresses in that K^+^ deficiency, and ABA treatment mainly increased the shaker K^+^ channel gene expression throughout the whole seedling, whereas NaCl and PEG treatment reduced the shaker K^+^ channel gene expression, especially in the roots (Figure 5). In particular, the K^+^ deficiency decreased the expression of 5 genes (*PpAKT1*, *PpAKT2*, *PpKC1*, *PpSKOR*, and *PpGORK*) in all the tested tissues, including leaves, stems, and roots, and *PpKAT1*in the aboveground parts and *PpSPIK* in the leaves. ABA treatment significantly reduced the expression of 3 genes (*PpAKT1*, *PpKC1*, and *PpSKOR*) throughout the whole seedlings, 2 genes (*PpKAT1* and *PpGORK*) in the shoots, and *PpAKT2* in the leaves. The expression of 4 genes (*PpAKT1*, *PpAKT2*, *PpKC1*, and *PpSKOR*) were enhanced in all the tested tissues and 2 genes (*PpKAT1* and *PpGORK*) in the roots under NaCl treatment, while 3 genes (*PpAKT1*, *PpKC1*, and *PpSKOR*) were increased throughout the whole seedlings, 2 genes (*PpKAT1* and *PpGORK*) in the leaves, and *PpGORK* in the roots and *PpAKT2* in the aboveground parts. Although the expression of *PpSPIK* was extremely low throughout the whole peach seedling, its expression changed little in all tested tissues under PEG treatment (Figure 5).

### 3.6. Electrophysiological Function of PpSKOR

**Figure 6:**
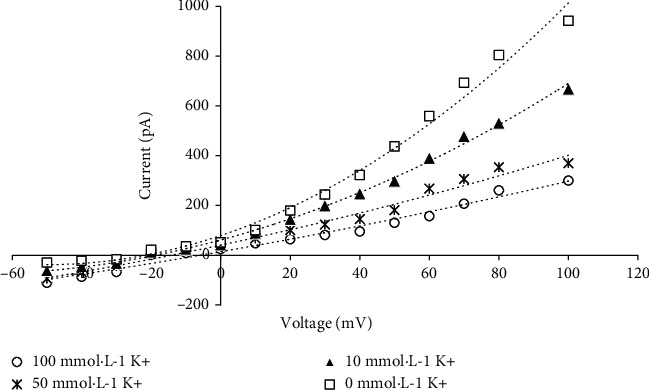
Curves of the current-voltage relation recorded by patch clamping system. Green fluorescence-labelled HEK293-T cells that transformed with pTracer-CMV3-*SKOR* were being detected by PCLAMP 10.0 device. The current signal was recorded by PCLAMP 10.0 and calculated by Sigma plot 11.0. The K^+^ concentration in the extracellular fluid was chosen as 0, 10, 50, and 100 mmol·L^−1^. Data are shown as the means recorded from 5 independent cells.

Considering that the overall expression amount of *PpSKOR* was relatively higher than that of the other shaker K^+^ channel genes, especially of the highest level in the roots, its expression was sensitive to all tested treatments, including K^+^ deficiency, NaCl, ABA, and PEG treatment (Figures 4 and 5). We further determined the electrophysiological function of *PpSKOR* by patch clamping system. The results revealed that cells expressing pTracer-CMV3-*SKOR* possessed outward-rectifying currents (Figure 6). Notably, the highest currents were recorded when the extracellular K^+^ concentration was 0 mmol·L^−1^, and the outward-rectifying currents decreased alongside with the increase of the extracellular K^+^ concentration (Figure 6). Moreover, the capacity of PpSKOR channel was activated when the cell membrane voltage was set at -20 mV, and the intensity of the outward-rectifying currents were increased when the voltage was more positive (Figure 6).

**Figure 7:**
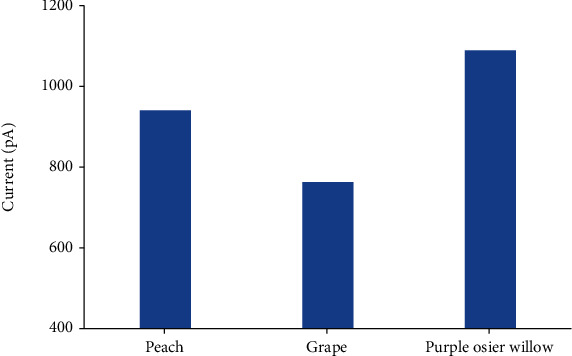
Current intensity comparison of SKOR channel homologs from peach, grape, and purple osier willow. The electrophysiological function of peach PpSKOR, grape VviSKOR [30], and purple osier willow SpuSKOR [9] was verified by patch clamping system. The current signal was recorded when the K^+^ concentration was set at 0 mmol·L^−1^ and the voltage was set at 100 mV. Data are shown as the means recorded from 5 independent cells.

Recently, the electrophysiological function of SpuSKOR [9] and VviSKOR [30] has been determined by patch clamping system. In this study, we further compared the intensity of outward currents among SKOR homologs of peach, grape, and purple osier willow. When the K^+^ concentration in the extracellular fluid was set at 0 mmol·L^−1^ and the cell membrane voltage was set at 100 mV, the outward current intensity of peach PpSKOR was higher than that of grape VviSKOR but lower than that of SpuSKOR from purple osier willow (Figure 7).

## 4. Discussion

K^+^ fertilizer plays a key role in tree growth, flowering, fruit quality, and yield [23–25, 28]. However, molecular mechanisms towards K^+^ nutrition in fruit trees are largely unclear, especially in peach.

In plants, the structures of the shaker K^+^ channels, including KAT, AKT, KC, SPIK, SKOR, and GORK types, are highly conserved and similar to that of *Drosophila* [8, 11, 32]. In this present study, the amino acid sequences of the shaker K^+^ channel proteins from 6 plant species mentioned above shared an extremely high identity in the conserved domains or regions (Supplemental Figure 1) and, again, support the proposition that the shaker K^+^ channel domains are highly conserved during long-distance evolution. According to the phylogenetic tree, the peach shaker K^+^ channels were classified into 5 subfamilies, I-V, which is consistent with the classification of *Arabidopsis* and pear shaker K^+^ channel proteins [1, 8, 11, 32]. Notably, the peach shaker K^+^ channel proteins are tightly clustered with pear homologs in the phylogenetic tree, implying that peach and pear belonging to the same *Rosaceae* may possess a closer evolution distance than the other 4 annual plants, including *Arabidopsis*, rice, sorghum, and maize (Figure 2).

Notably, tissue-/organ-specific expression patterns of the shaker K^+^ channel genes may reflect their precise functions during plant growth and development [1, 8, 9, 32]. In this study, *PpSPIK* was absolutely expressed in mature whole flowers (Figure 4), including pollen, which was consistent with the previous report that *AtSPIK* was majorly expressed in pollen and mediated inward K^+^ influx into the growing tube [33, 34]. We speculate that *PpSPIK* may play similar roles in peach tube development that needs further functional determination. In *Arabidopsis*, *AtKAT1* was mainly expressed in leaf guard cells and functioned as an inward-rectifying K^+^ channel [35], while *AtGORK* was mainly expressed in the leaves and functioned as an outwardly-rectifying K^+^ channel of the guard cell membrane [36, 37]. Together, these two channels contribute to stoma movement and K^+^ balance in *Arabidopsis* [35–37]. Consistently, both *PpKAT1* and *PpGORK* were dominantly expressed in peach leaves, including young and mature leaves, implying that these two channel genes may also be involved in stoma movement in peach leaves. In addition, SKOR as outward-rectifying K^+^ channel was famous for the long-distance transportation of K^+^ ions through the xylem in plants [9, 38–41].

Similar expression profiles may reflect physiological functions. In this present study, peach *PpSKOR* was mainly expressed in the roots and also be observed in the leaves, phloem, and flowers, which was in line with the previous reports of *SKOR* homologous genes from muskmelon, osier willow, and *Z*. *xanthoxylum* [9, 40, 42]. However, *SKOR* was specifically expressed in the roots of *Arabidopsis* and rice [38, 39]. Further patch clamping analysis revealed that PpSKOR demonstrated K^+^ efflux current and voltage-dependent gated channel activity, which belong to the characteristics of outward-rectifying K^+^ channels (Figure 6). These findings are in accordance with grape VviSKOR [30] and purple osier willow SpuSKOR [9] that are being verified by patch clamping systems via HEK 293-T cells but also in line with *Arabidopsis* AtSKOR [38] and muskmelon CmSKOR [40] that are being determined by *Xenopus* oocytes and double electrode voltage clamp systems. Although similar outward current characteristics of SKOR homologs are being observed, the current intensity is different among distinct plant species, and the channel activity of PpSKOR was higher than that of grape VviSKOR but lower than that of SpuSKOR from purple osier willow (Figure 7), implying that the physiological function and regulatory mechanisms of SKOR homologous channels are not only specific but also complex, especially in woody plants. Nonetheless, we consider that PpSKOR is an indispensable outward-rectifying K^+^ channel in peach trees.

Shaker K^+^ channels play an important role in K^+^ homeostasis, osmotic regulation, and proton regulation and are regulated by abiotic stresses [1, 9, 32, 38–42]. In this study, all peach shaker K^+^ channel genes were sensitive to abiotic stresses, whose expression level was changed in at least one tested tissue, except for *PpSPIK* that changed little under each treatment (Figure 5). Mainly expressed in the roots, *PpAKT1*, *PpKC1*, and *PpSKOR* were the most sensitive shaker K^+^ channel genes in peach, whose expression was prone to be regulated in all tested tissues under each treatment. These findings were consistent with the previous studies in *Arabidopsis* [35–38], rice [39], *Z*. *xanthoxylum* [42], muskmelon [40], and osier pillow [9].

Nonetheless, these 7 shaker K^+^ channel genes may play distinct and precise roles during peach tree growth and development, which lays a molecular basis for further functional studies of the shaker-type K^+^ channel genes in fruit trees.

## 5. Conclusion

The seven peach shaker-type K^+^ channel proteins were tightly clustered with pear homologs in the phylogenetic tree. The peach shaker K^+^ channel genes were differentially expressed in distinct tissues, and K^+^ deficiency and ABA treatment mainly increased their gene expression throughout the whole seedling, whereas NaCl and PEG treatment reduced their gene expression. PpSKOR is a typical voltage-dependent outward-rectifying K^+^ channel in peach. This study lays a molecular basis for functional studies of the shaker-type K^+^ channels in peach.

## Figures and Tables

**Table 1:** Basic information of the peach shaker K^+^ channel genes.

Gene name	Locus ID	Chr	Gene location	Intron no.	Subgroup	Protein (aa)	pI	TM	Aliphatic index	GRAVY
*PpKAT1*	Prupe.4G080000	4	3881434..3886944 forward	10	Group II	776	6.26	6	85.96	-0.24
*PpSPIK*	Prupe.1G472600	1	39291448..39296784 reverse	11	Group I	897	6.47	6	94.39	-0.12
*PpAKT1*	Prupe.7G237400	7	20553574..20560807 reverse	10	Group I	890	4.88	6	127.72	0.728
*PpAKT2*	Prupe.1G572200	1	46649721..46658449 forward	12	Group III	843	7.55	6	94.62	-0.18
*PpKC1*	Prupe.1G464600	1	38774545..38780568 forward	10	Group IV	627	6.65	6	98.55	-0.01
*PpSKOR*	Prupe.5G237000	5	17914373-17920240 forward	10	Group V	750	6.22	6	94.94	-0.19
*PpGORK*	Prupe.3G164900	3	18394189..18405879 reverse	12	Group V	831	6.02	6	98.42	-0.14

**Table 2:** Subcellular localization prediction of the peach shaker K^+^ channel proteins*^a^*.

Gene	Plasma membrane	Endoplasmic reticulum membrane	Cytosol	Microbody	Nucleus	Mitochondrial inner membrane	Chloroplast membrane	Golgi body
*PpKAT1*	64.30%	14.28%	—	7.14%	7.14%	7.14%	—	—
*PpSPIK*	64.30%	21.42%	7.14%	—	7.14%	—	—	—
*PpAKT1*	100%	—	—	—	—	—	—	—
*PpAKT2*	57.16%	21.42%	14.28%	—	7.14	—	—	—
*PpKC1*	78.58%	7.14%	—	7.14%	—	7.14%	—	—
*PpSKOR*	64.30%	21.42%	14.28%	—	—	—	—	—
*PpGORK*	50%	—	7.14%	7.14%	—	—	28.58%	7.14%

*^a^* Indicates no detection.

**Table 3:** *Cis*-acting elements analysis in the promoter regions of the peach shaker K^+^ channel genes*^a^*.

*Cis*-acting elements	Proposed functions	*PpKAT1*	*PpSPIK*	*PpAKT1*	*PpAKT2*	*PpKC1*	*PpSKOR*	*PpGORK*
GT1-motif	Light response	6	6	7	6	7	8	7
ARE	Anaerobic induction	1	1	1	1	1	1	1
ABRE	Abscisic acid responsive	1	1	1	1	1	1	1
TGACG-motif	Methyl jasmonate	2	2	—	—	3	2	2
AACA_motif	Endosperm expression	—	1	1	1	1	1	—
MBS	Drought inducibility	—	1	—	1	1	—	1
TATC-box	Gibberellin responsive	1	1	—	—	—	—	1
O2-site	Zein metabolism	1	—	1	—	—	—	1
AuxRR-core	Auxin responsive	—	—	—	1	—	—	3
TCA-element	Salicylic acid responsive	—	—	—	1	2	1	—
CAT-box	Meristem expression	1	—	1	—	—	1	—
TC-rich repeats	Wound responsive	—	—	1	—	—	—	—
MYB	Flavonoid biosynthesis	—	1	—	—	—	—	—
LTR	Low temperature	—	1	—	—	—	—	—
CARE	Metabolism regulation	—	—	—	—	—	1	—
TC-rich repeats	Defence and stress	—	—	—	1	—	—	—

*^a^* Indicates no detection.

## Data Availability

The data used to support the findings of this study are included within the article.

## Supplementary Materials

Supplementary Materials Supplemental Figure 1: amino acid alignment of highly conserved domains of plant shaker K^+^ channels. Supplemental Table 1: gene ID of plant shaker K^+^ channels used for phylogenetic tree construction. Supplemental Table 2: specific primers used in this study.
