# Supplementary material for: Genome-Wide Identification and Characterization of the Shaker-Type K+ Channel Genes in Prunus persica (L.) Batsch
Source: Int J Genomics. 2022 Mar 9;2022:5053838. doi: 10.1155/2022/5053838 (PMC8926527; doi:10.1155/2022/5053838)
Supplement: Supplementary Materials — Supplemental Figure 1: amino acid alignment of highly conserved domains of plant shaker K+ channels. Supplemental Table 1: gene ID of plant shaker K+ channels used for phylogenetic tree construction. Supplemental Table 2: specific primers used in this study. [file 5053838.f1.zip › Supplemental Table 2 (1).docx]

**Supplemental Table 2** Specific primers used in this study..

| Name | Primer (5’ to 3’) | Amplicon size (bp) |
| --- | --- | --- |
| *PpKAT1* | F: TTTACGACTCCGCAGCAGTT  R: CCCTTCTTGCCCCTTCAGTT | 106 |
| *PpSPIK* | F: AAAGGAGCGGATGCTGACAA  R: TCACGGGCATAGGGAGGTAA | 206 |
| *PpAKT1* | F: CGTCCCAGGACTGCTCATTT  R: AACAAGTCTTTCTCGCCGGT | 136 |
| *PpAKT2* | F: CCTTCCTCCTTTGGGTGCTT  R: CACCACGTTGTCTGCTAGGT | 205 |
| *PpKC1* | F: AAGCAGCATCTTACCGGCTT  R: ATCAACGGGCATGAGAGACC | 199 |
| *PpSKOR* | F: GCCCTCCGCTACCTAAAGTC  R: CTCGGCTCAGACGTATCCAC | 127 |
| *PpGORK* | F: AGGTTTGATCCGTGCTGGAG  R: AGAAACTCGATCGTTGCCGT | 196 |
| *PpUBI* | F: AGGCTAAGATCCAAGACAAAGAG  R: CCACGAAGACGAAGCACTAAG | 145 |
| pTracer-CMV3-*SKOR* | F: GCGGTTTAAACATGCATGGAGCTGAGAGAAG  R: GAGGCGGCCGCTTACAACTGCGAAGCTCTAG | 2250 |
