# Supplementary material for: Genome-Wide Identification and Characterization of the Shaker-Type K+ Channel Genes in Prunus persica (L.) Batsch
Source: Int J Genomics. 2022 Mar 9;2022:5053838. doi: 10.1155/2022/5053838 (PMC8926527; doi:10.1155/2022/5053838)
Supplement: Supplementary Materials — Supplemental Figure 1: amino acid alignment of highly conserved domains of plant shaker K+ channels. Supplemental Table 1: gene ID of plant shaker K+ channels used for phylogenetic tree construction. Supplemental Table 2: specific primers used in this study. [file 5053838.f1.zip › Supplemental Table 1 (1).docx]

| Gene | Gene ID | protein length (aa) |
| --- | --- | --- |
| *AtAKT1* | At2g26650 | 857 |
| *AtSPIK* | At2g25600 | 888 |
| *AtAKT5* | At4g32500 | 880 |
| *AtKAT1* | At5g46240 | 677 |
| *AtKAT2* | At4g18290 | 697 |
| *AtAKT2* | At4g22200 | 802 |
| *AtKC1* | At4g32650 | 662 |
| *AtSKOR* | At3g02850 | 828 |
| *AtGORK* | At5g37500 | 820 |
| *PbrGORK* | Pbr016651.1 | 1481 |
| *PbrKAT1* | Pbr039581.1 | 771 |
| *PbrSPIK* | Pbr025424.1 | 775 |
| *PbrAKT1* | Pbr001827.1 | 879 |
| *PbrKC2* | Pbr002265.1 | 587 |
| *PbrAKT2* | Pbr026531.1 | 840 |
| *PbrKC1* | Pbr021268.1 | 620 |
| *PbrSKOR* | Pbr022827.1 | 839 |
| OsKAT2 | LOC_Os01g11250.1 | 568 |
| *OsAKT1.2* | LOC_Os07g07910.1 | 891 |
| *OsSKOR* | LOC_Os06g14030.1 | 858 |
| *OsAKT2* | LOC_Os05g35410.1 | 703 |
| *OsGORK* | LOC_Os04g36740.1 | 722 |
| *OsAKT1.1* | LOC_Os01g45990.1 | 935 |
| *OsKAT1* | LOC_Os01g55200.1 | 502 |
| *OsKAT3* | LOC_Os02g14840.1 | 718 |
| *OsKC1.2* | LOC_Os04g02720.1 | 368 |
| *OsKC1.3* | LOC_Os06g14310.1 | 591 |
| *OsKC1.1* | LOC_Os01g52070.1 | 593 |
| *SbKAT1* | *Sobic.003G300600.1* | 530 |
| *SbKAT2* | *Sobic.004G107500.1* | 729 |
| *ZmKAT1* | *Zm00008a021554* | 738 |
| *ZmKAT2* | *Zm00008a033192* | 505 |

**Supplemental Table 1** Gene ID of plant Shaker K^+^ channels used for phylogenetic tree construction.
