# Supplementary material for: Genome-Wide Identification and Characterization of the Shaker-Type K+ Channel Genes in Prunus persica (L.) Batsch
Source: Int J Genomics. 2022 Mar 9;2022:5053838. doi: 10.1155/2022/5053838 (PMC8926527; doi:10.1155/2022/5053838)
Supplement: Supplementary Materials — Supplemental Figure 1: amino acid alignment of highly conserved domains of plant shaker K+ channels. Supplemental Table 1: gene ID of plant shaker K+ channels used for phylogenetic tree construction. Supplemental Table 2: specific primers used in this study. [file 5053838.f1.zip › Supplemental files.pdf]

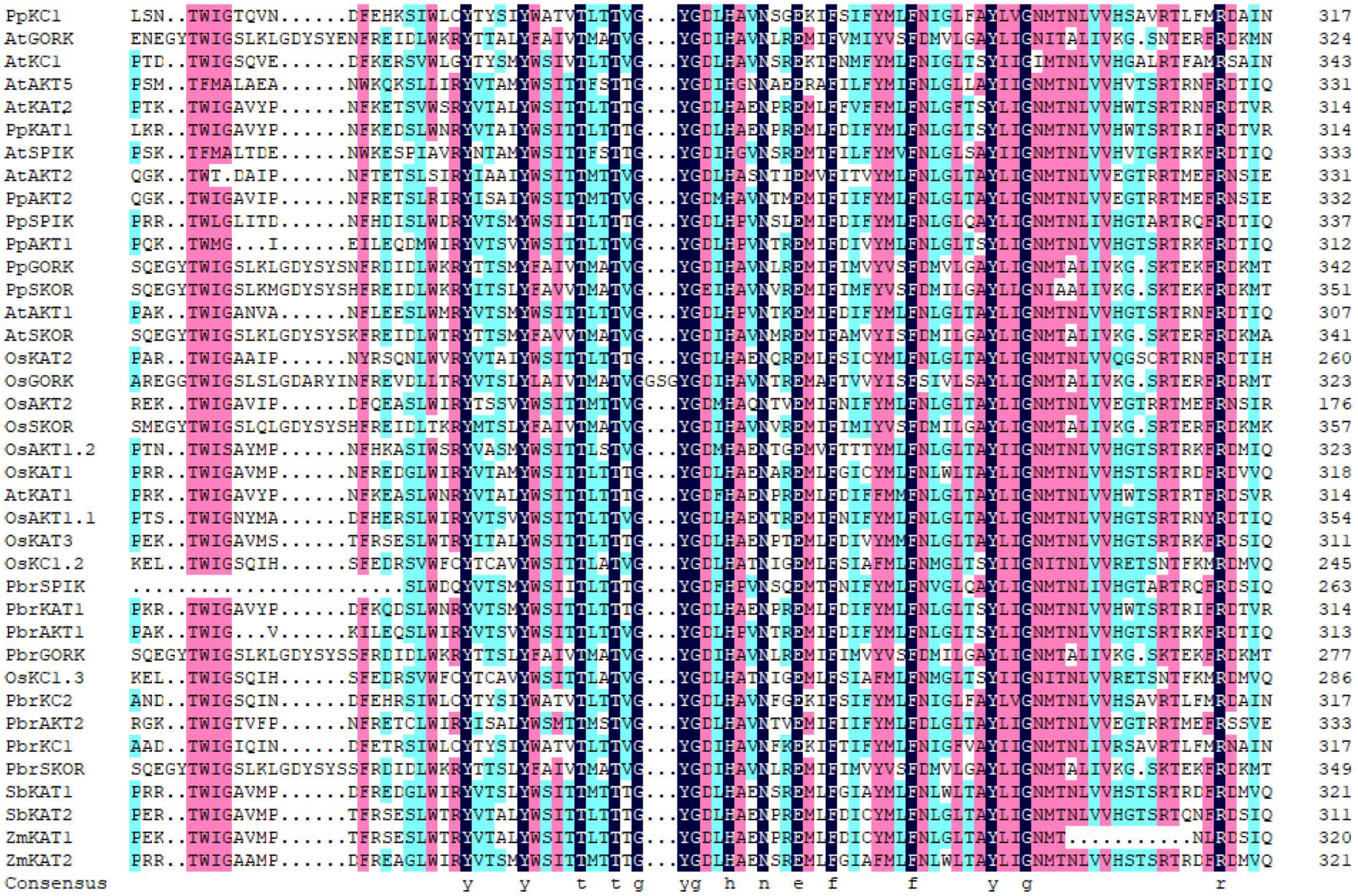
PpKC1 LSN..TWIGTQVN......DFEHKSIWLCYTYSIYWATVTLTTVG...YGDLHAVNSGEKIFSIFYMLFNIGLFAYLVGNMTNLVVHSAVRTLFMRDAIN 317
AtGORK ENEGYTWIGSLKLGDYSYENFREIDLWKRYTTALYFAIVTMATVG...YGDIHAVNLREMIFVMIYVSFDMVLGAYLIGNITALIVKG.SNTERFRDKMN 324
AtKC1 PTD..TWIGSQVE......DFKERSVWLGYTYSMYWSIVTLTTVG...YGDLHATNSREKTFNMFYMLFNIGLTSYIIGIMTNLVVHGALRTFAMRSAIN 343
AtAKT5 PSM..TFMALAEA......NWKQKSLLIRYVTAMYWSITTFSTTG...YGDIHGNNAEERAFILFYMIFNLGLLAYIIGNMTNLVVHVTSRTRNFRDTIQ 331
AtKAT2 PTK..TWIGAVYP......NFKETSVWSRYVTALYWSITTLTTTG...YGDLHAENPREMLFFVFFMLFNLGFTSYLIGNMTNLVVHWTSRTRNFRDTVR 314
PpKAT1 LKR..TWIGAVYP......NFKEDSLWNRYVTAIYWSITTLTTTG...YGDLHAENPREMLFDIFYMLFNLGLTSYLIGNMTNLVVHWTSRTRIFRDTVR 314
AtSPIK PSK..TFMALTDE......NWKESPIAVRYNTAMYWSITTFSTTG...YGDIHGVNSREMTFILFYMVFNLGLSAYIIGNMTNLVVHVTGRTRKFRDTIQ 333
AtAKT2 QGK..TWT.DAIP......NFTETSLSIRYIAAIYWSITTMTTVG...YGDLHASNTIEMVFITVYMLFNLGLTAYLIGNMTNLVVEGTRRTMEFRNSIE 331
PpAKT2 QGK..TWIGAVIP......NFRETSLRIRYISAIYWSITTMTTVG...YGDMHAVNTMEMIFIIFYMLFNLGLTAYLIGNMTNLVVEGTRRTMEFRNSIE 332
PpSPIK PRR..TWLGLITD......NFHDISLWDRYVTSMYWSIITLTTTG...YGDLHPVNSLEMIFDIFYMLFNLGLQAYLIGNMTNLIVHGTARTRQFRDTIQ 337
PpAKT1 PQK..TWMG...I......EILEQDMWIRYVTSVYWSITTLTTVG...YGDLHPVNTREMIFDIVYMLFNLGLTSYLIGNMTNLVVHGTSRTRKFRDTIQ 312
PpGORK SQEGYTWIGSLKLGDYSYSNFRDIDLWKRYTTSMYFAIVTMATVG...YGDIHAVNLREMIFIMVYVSFDMVLGAYLIGNMTALIVKG.SKTEKFRDKMT 342
PpSKOR SQEGYTWIGSLKMGDYSYSHFREIDLWKRYITSLYFAVVTMATVG...YGEIHAVNVREMIFIMFYVSFDMILGAYLLGNIAALIVKG.SKTEKFRDKMT 351
AtAKT1 PAK..TWIGANVA......NFLEESLWMRYVTSMYWSITTLTTVG...YGDLHPVNTKEMIFDIFYMLFNLGLTAYLIGNMTNLVVHGTSRTRNFRDTIQ 307
AtSKOR SQEGYTWIGSLKLGDYSYSKFREIDLWTRYTTSMYFAVVTMATVG...YGDIHAVNMREMIFAMVYISFDMILGAYLIGNMTALIVKG.SKTERFRDKMA 341
OsKAT2 PAR..TWIGAAIP......NYRSQNLWVRYVTAIYWSITTLTTTG...YGDLHAENQREMLFSICYMLFNLGLTAYLIGNMTNLVVQGSCRTRNFRDTIH 260
OsGORK AREGGTWIGSLSLGDARYINFREVDLLTRYVTSLYLAIVTMATVGGSGYGDIHAVNTREMAFTVVYISFSIVLSAYLIGNMTALIVKG.SRTERFRDRMT 323
OsAKT2 REK..TWIGAVIP......DFQEASLWIRYTSSVYWSITTMTTVG...YGDMHAQNTVEMIFNIFYMLFNLGLTAYLIGNMTNLVVEGTRRTMEFRNSIR 176
OsSKOR SMEGYTWIGSLQLGDYSYSHFREIDLTKRYMTSLYFAIVTMATVG...YGDIHAVNVREMIFIMIYVSFDMIIGAYLIGNMTALIVKG.SRTERFRDKMK 357
OsAKT1.2 PTN..TWISAYMP......NFHKASIWSRYVASMYWSITTLSTVG...YGDMHAENTGEMVFTTTYMLFNLGLTAYIIGNMTNLVVHGTSRTRKFRDMIQ 323
OsKAT1 PRR..TWIGAVMP......NFREDGLWIRYVTAMYWSITTLTTTG...YGDLHAENAREMLFGICYMLFNLWLTAYLIGNMTNLVVHSTSRTRDFRDVVQ 318
AtKAT1 PRK..TWIGAVYP......NFKEASLWNRYVTALYWSITTLTTTG...YGDFHAENPREMLFDIFFMMFNLGLTAYLIGNMTNLVVHWTSRTRTFRDSVR 314
OsAKT1.1 PTS..TWIGNYMA......DFHERSLWIRYVTSVYWSITTLTTVG...YGDLHAENTREMIFNIFYMLFNLGLTAYLIGNMTNLVVHGTSRTRNYRDTIQ 354
OsKAT3 PEK..TWIGAVMS......TFRSESLWTRYITALYWSITTLTTTG...YGDLHAENPTEMLFDIVYMMFNLGLTAYLIGNMTNLVVHGTSRTRKFRDSIQ 311
OsKC1.2 KEL..TWIGSQIH......SFEDRSVWFCYTCAVYWSITTLATVG...YGDLHATNIGEMLFSIAFMLFNMGLTSYIIGNITNLVVRETSNTFKMRDMVQ 245
PbrSPIK .......................SLWDQYVTSMYWSIITLTTTG...YGDFHPVNSQEMTFNIFYMLFNVGLQAYLIGNMTNLIVHGTARTRQFRDSIQ 263
PbrKAT1 PKR..TWIGAVYP......DFKQDSLWNRYVTSMYWSITTLTTTG...YGDLHAENPREMLFDIFYMLFNLGLTSYLIGNMTNLVVHWTSRTRIFRDTVR 314
PbrAKT1 PAK..TWIG...V......KILEQSLWIRYVTSVYWSITTLTTVG...YGDLHPVNTREMIFDIFYMLFNLGLTSYLIGNMTNLVVHGTSRTRKFRDTIQ 313
PbrGORK SQEGYTWIGSLKLGDYSYSSFRDIDLWKRYTTSLYFAIVTMATVG...YGDIHAVNLREMIFIMVYVSFDMILGAYLIGNMTALIVKG.SKTEKFRDKMT 277
OsKC1.3 KEL..TWIGSQIH......SFEDRSVWFCYTCAVYWSITTLATVG...YGDLHATNIGEMLFSIAFMLFNMGLTSYIIGNITNLVVRETSNTFKMRDMVQ 286
PbrKC2 AND..TWIGSQIN......DFEHRSIWLCYTYSIYWATVTLTTVG...YGDLHAVNFGEKIFSIFYMLFNIGLFAYLVGNMTNLVVHSAVRTLFMRDAIN 317
PbrAKT2 RGK..TWIGTVFP......NFRETCLWIRYISALYWSMTTMSTVG...YGDLHAVNTVEMIFIIFYMLFDLGLTAYLIGNMTNLVVEGTRRTMEFRSSVE 333
PbrKC1 AAD..TWIGIQIN......DFETRSIWLCYTYSIYWATVTLTTVG...YGDIHAVNFKEKIFTIFYMLFNIGFVAYIIGNMTNLIVRSAVRTLFMRNAIN 317
PbrSKOR SQEGYTWIGSLKLGDYSYSSFRDIDLWKRYTTSLYFAIVTMATVG...YGDIHAVNLREMIFIMVYVSFDMVLGAYLIGNMTALIVKG.SKTEKFRDKMT 349
SbKAT1 PRR..TWIGAVMP......DFREDGLWIRYVTSLYWSITTMTTTG...YGDLHAENSREMLFGIAYMLFNLWLTAYLIGNMTNLVVHSTSRTRDFRDMVQ 321
SbKAT2 PER..TWIGAVMP......TFRSESLWTRYVTALYWSITTLTTTG...YGDLHAENPREMLFDICYMLFNLGLTAYLIGNMTNLVVHGTSRTQNFRDSIQ 311
ZmKAT1 PEK..TWIGAVMP......TFRSESLWTRYVTALYWSITTLTTTG...YGDLHAENPREMLFDICYMLFNLGLTAYLIGNMT...........NLRDSIQ 320
ZmKAT2 PRR..TWIGAAMP......DFREAGLWIRYVTSMYWSITTMTTTG...YGDLHAENSREMLFGIAFMLFNLWLTAYLIGNMTNLVVHSTSRTRDFRDMVQ 321
Consensus y y t t g yg h n e f f y g r

Supplemental Table 1 Gene ID of plant Shaker K$^+$ channels used for phylogenetic tree construction.

Gene	Gene ID	protein length (aa)
AtAKT1	At2g26650	857
AtSPIK	At2g25600	888
AtAKT5	At4g32500	880
AtKAT1	At5g46240	677
AtKAT2	At4g18290	697
AtAKT2	At4g22200	802
AtKC1	At4g32650	662
AtSKOR	At3g02850	828
AtGORK	At5g37500	820
PbrGORK	Pbr016651.1	1481
PbrKAT1	Pbr039581.1	771
PbrSPIK	Pbr025424.1	775
PbrAKT1	Pbr001827.1	879
PbrKC2	Pbr002265.1	587
PbrAKT2	Pbr026531.1	840
PbrKC1	Pbr021268.1	620
PbrSKOR	Pbr022827.1	839
OsKAT2	LOC_Os01g11250.1	568
OsAKT1.2	LOC_Os07g07910.1	891
OsSKOR	LOC_Os06g14030.1	858
OsAKT2	LOC_Os05g35410.1	703
OsGORK	LOC_Os04g36740.1	722
OsAKT1.1	LOC_Os01g45990.1	935
OsKAT1	LOC_Os01g55200.1	502
OsKAT3	LOC_Os02g14840.1	718
OsKC1.2	LOC_Os04g02720.1	368
OsKC1.3	LOC_Os06g14310.1	591
OsKC1.1	LOC_Os01g52070.1	593
SbKAT1	*Sobic.003G300600.1*	530
SbKAT2	*Sobic.004G107500.1*	729
ZmKAT1	*Zm00008a021554*	738
ZmKAT2	*Zm00008a033192*	505

Supplemental Table 2 Specific primers used in this study..

Name	Primer (5' to 3')	Amplicon size (bp)
PpKAT1	F: TTTACGACTCCGCAGCAGTT<br>R: CCCTTCTTGCCCCTTCAGTT	106
PpSPIK	F: AAAGGAGCGGATGCTGACAA<br>R: TCACGGGCATAGGGAGGTAA	206
PpAKT1	F: CGTCCCAGGACTGCTCATTT<br>R: AACAAGTCTTTCTCGCCGGT	136
PpAKT2	F: CCTTCCTCCTTTGGGTGCTT<br>R: CACCACGTTGTCTGCTAGGT	205
PpKC1	F: AAGCAGCATCTTACCGGCTT<br>R: ATCAACGGGCATGAGAGACC	199
PpSKOR	F: GCCCTCCGCTACCTAAAGTC<br>R: CTCGGCTCAGACGTATCCAC	127
PpGORK	F: AGGTTTGATCCGTGCTGGAG<br>R: AGAAACTCGATCGTTGCCGT	196
PpUBI	F: AGGCTAAGATCCAAGACAAAGAG<br>R: CCACGAAGACGAAGCACTAAG	145
pTracer-CMV3-*SKOR*	F: GCGGTTTAAACATGCATGGAGCTGAGAGAAG<br>R: GAGGCGGCCGCTTACAACTGCGAAGCTCTAG	2250
