# Supplementary material for: Genome-Wide Identification and Characterization of the Shaker-Type K+ Channel Genes in Prunus persica (L.) Batsch
Source: Int J Genomics. 2022 Mar 9;2022:5053838. doi: 10.1155/2022/5053838 (PMC8926527; doi:10.1155/2022/5053838)
Supplement: Supplementary Materials — Supplemental Figure 1: amino acid alignment of highly conserved domains of plant shaker K+ channels. Supplemental Table 1: gene ID of plant shaker K+ channels used for phylogenetic tree construction. Supplemental Table 2: specific primers used in this study. [file 5053838.f1.zip › Supplemental Figure 1 (1).pdf]

```
PpKC1         LSN..TWIGTQVN......DFEHKSIWLCYTYSIYWATVTLTTVG...YGDLHAVNSGEKIFSIFYMLFNIGLFAYLVGNMTNLVVHSAVRTLFMRDAIN   317
AtGORK        ENEGYTWIGSLKLGDYSYENFREIDLWKRYTTALYFAIVTMATVG...YGDIHAVNLREMIFVMIYVSFDMVLGAYLIGNITALIVKG.SNTERFRDKMN   324
AtKC1         PTD..TWIGSQVE......DFKERSVWLGYTYSMYWSIVTLTTVG...YGDLHAVNSREKTFNMFYMLFNIGLTSYIIGIMTNLVVHGALRTFAMRSAIN   343
AtAKT5        PSM..TFMALAEA......NWKQKSLLIRYVTAMYWSITTFSTTG...YGDIHGNNAEERAFILFYMIFNLGLLAYIIGNMTNLVVHVTSRTRNFRDTIQ   331
AtKAT2        PTK..TWIGAVYP......NFKETSVWSRYVTALYWSITTLTTTG...YGDLHAENPREMLFFVFFMLFNLGFTSYLIGNMTNLVVHWTSRTRNFRDTVR   314
PpKAT1        LKR..TWIGAVYP......NFKEDSLWNRYVTAIYWSITTLTTTG...YGDLHAENPREMLFDIFYMLFNLGLTSYLIGNMTNLVVHWTSRTRIFRDTVR   314
AtSPIK        PSK..TFMALTDE......NWKESPIAVRYNTAMYWSITTFSTTG...YGDIHGVNSREMTFILFYMVFNLGLSAYIIGNMTNLVVHVTGRTRKFRDTIQ   333
AtAKT2        QGK..TWT.DAIP......NFTETSLSIRYIAAIYWSITTMTTVG...YGDLHASNTIEMVFITVYMLFNLGLTAYLIGNMTNLVVEGTRRTMEFRNSIE   331
PpAKT2        QGK..TWIGAVIP......NFRETSLRIRYISAIYWSITTMTTVG...YGDMHAVNTMEMIFIIFYMLFNLGLTAYLIGNMTNLVVEGTRRTMEFRNSIE   332
PpSPIK        PRR..TWLGLITD......NFHDISLWDRYVTSMYWSIITLTTTG...YGDLHPVNSLEMIFDIFYMLFNLGLQAYLIGNMTNLIVHGTARTRQFRDTIQ   337
PpAKT1        PQK..TWMG...I......EILEQDMWIRYVTSVYWSITTLTTVG...YGDLHPVNTREMIFDIVYMLFNLGLTSYLIGNMTNLVVHGTSRTRKFRDTIQ   312
PpGORK        SQEGYTWIGSLKLGDYSYSNFRDIDLWKRYTTSMYFAIVTMATVG...YGDIHAVNLREMIFIMVYVSFDMVLGAYLIGNMTALIVKG.SKTEKFRDKMT   342
PpSKOR        SQEGYTWIGSLKMGDYSYSHFREIDLWKRYITSLYFAVVTMATVG...YGEIHAVNVREMIFIMFYVSFDMILGAYLLGNIAALIVKG.SKTEKFRDKMT   351
AtAKT1        PAK..TWIGANVA......NFLEESLWMRYVTSMYWSITTLTTVG...YGDLHPVNTKEMIFDIFYMLFNLGLTAYLIGNMTNLVVHGTSRTRNFRDTIQ   307
AtSKOR        SQEGYTWIGSLKLGDYSYSKFREIDLWTRYTTSMYFAVVTMATVG...YGDIHAVNMREMIFAMVYISFDMILGAYLIGNMTALIVKG.SKTERFRDKMA   341
OsKAT2        PAR..TWIGAAIP......NYRSQNLWVRYVTAIYWSITTLTTTG...YGDLHAENQREMLFSICYMLFNLGLTAYLIGNMTNLVVQGSCRTRNFRDTIH   260
OsGORK        AREGGTWIGSLSLGDARYINFREVDLLTRYVTSLYLAIVTMATVGGSGYGDIHAVNTREMAFTVVYISFSIVLSAYLIGNMTALIVKG.SRTERFRDRMT   323
OsAKT2        REK..TWIGAVIP......DFQEASLWIRYTSSVYWSITTMTTVG...YGDMHAQNTVEMIFNIFYMLFNLGLTAYLIGNMTNLVVEGTRRTMEFRNSIR   176
OsSKOR        SMEGYTWIGSLQLGDYSYSHFREIDLTKRYMTSLYFAIVTMATVG...YGDIHAVNVREMIFIMIYVSFDMILGAYLIGNMTALIVKG.SRTERFRDKMK   357
OsAKT1.2      PTN..TWISAYMP......NFHKASIWSRYVASMYWSITTLSTVG...YGDMHAENTGEMVFTTTYMLFNLGLTAYIIGNMTNLVVHGTSRTRKFRDMIQ   323
OsKAT1        PRR..TWIGAVMP......NFREDGLWIRYVTAMYWSITTLTTTG...YGDLHAENAREMLFGICYMLFNLWLTAYLIGNMTNLVVHSTSRTRDFRDVVQ   318
AtKAT1        PRK..TWIGAVYP......NFKEASLWNRYVTALYWSITTLTTTG...YGDFHAENPREMLFDIFFMMFNLGLTAYLIGNMTNLVVHWTSRTRTFRDSVR   314
OsAKT1.1      PTS..TWIGNYMA......DFHERSLWIRYVTSVYWSITTLTTVG...YGDLHAENTREMIFNIFYMLFNLGLTAYLIGNMTNLVVHGTSRTRNYRDTIQ   354
OsKAT3        PEK..TWIGAVMS......TFRSESLWTRYITALYWSITTLTTTG...YGDLHAENPTEMLFDIVYMMFNLGLTAYLIGNMTNLVVHGTSRTRKFRDSIQ   311
OsKC1.2       KEL..TWIGSQIH......SFEDRSVWFCYTCAVYWSITTLATVG...YGDLHATNIGEMLFSIAFMLFNMGLTSYIIGNITNLVVRETSNTFKMRDMVQ   245
PbrSPIK       ........................SLWDQYVTSMYWSIITLTTTG...YGDFHPVNSQEMTFNIFYMLFNVGLQAYLIGNMTNLIVHGTARTRQFRDSIQ   263
PbrKAT1       PKR..TWIGAVYP......DFKQDSLWNRYVTSMYWSITTLTTTG...YGDLHAENPREMLFDIFYMLFNLGLTSYLIGNMTNLVVHWTSRTRIFRDTVR   314
PbrAKT1       PAK..TWIG...V......KILEQSLWIRYVTSVYWSITTLTTVG...YGDLHPVNTREMIFDIFYMLFNLGLTSYLIGNMTNLVVHGTSRTRKFRDTIQ   313
PbrGORK       SQEGYTWIGSLKLGDYSYSSFRDIDLWKRYTTSLYFAIVTMATVG...YGDIHAVNLREMIFIMVYVSFDMILGAYLIGNMTALIVKG.SKTEKFRDKMT   277
OsKC1.3       KEL..TWIGSQIH......SFEDRSVWFCYTCAVYWSITTLATVG...YGDLHATNIGEMLFSIAFMLFNMGLTSYIIGNITNLVVRETSNTFKMRDMVQ   286
PbrKC2        AND..TWIGSQIN......DFEHRSIWLCYTYSIYWATVTLTTVG...YGDLHAVNFGEKIFSIFYMLFNIGLFAYLVGNMTNLVVHSAVRTLFMRDAIN   317
PbrAKT2       RGK..TWIGTVFP......NFRETCLWIRYISALYWSMTTMSTVG...YGDLHAVNTVEMIFIIFYMLFDLGLTAYLIGNMTNLVVEGTRRTMEFRSSVE   333
PbrKC1        AAD..TWIGIQIN......DFETRSIWLCYTYSIYWATVTLTTVG...YGDIHAVNFKEKIFTIFYMLFNIGFVAYIIGNMTNLIVRSAVRTLFMRNAIN   317
PbrSKOR       SQEGYTWIGSLKLGDYSYSSFRDIDLWKRYTTSLYFAIVTMATVG...YGDIHAVNLREMIFIMVYVSFDMVLGAYLIGNMTALIVKG.SKTEKFRDKMT   349
SbKAT1        PRR..TWIGAVMP......DFREDGLWIRYVTSLYWSITTMTTTG...YGDLHAENSREMLFGIAYMLFNLWLTAYLIGNMTNLVVHSTSRTRDFRDMVQ   321
SbKAT2        PER..TWIGAVMP......TFRSESLWTRYVTALYWSITTLTTTG...YGDLHAENPREMLFDICYMLFNLGLTAYLIGNMTNLVVHGTSRTQNFRDSIQ   311
ZmKAT1        PEK..TWIGAVMP......TFRSESLWTRYVTALYWSITTLTTTG...YGDLHAENPREMLFDICYMLFNLGLTAYLIGNMT...........NLRDSIQ   320
ZmKAT2        PRR..TWIGAAMP......DFREAGLWIRYVTSMYWSITTMTTTG...YGDLHAENSREMLFGIAFMLFNLWLTAYLIGNMTNLVVHSTSRTRDFRDMVQ   321
Consensus                                    y    y    t  t g   yg  h  n  e  f      f      y  g                r
```
